# Phytohormones Regulate Both “Fish Scale” Galls and Cones on *Picea koraiensis*

**DOI:** 10.3389/fpls.2020.580155

**Published:** 2020-11-27

**Authors:** Mingyue Jia, Qilong Li, Juan Hua, Jiayi Liu, Wei Zhou, Bo Qu, Shihong Luo

**Affiliations:** ^1^ College of Bioscience and Biotechnology, Shenyang Agricultural University, Shenyang, China; ^2^ Key Laboratory of Biological Invasions and Global Changes, Shenyang Agricultural University, Shenyang, China

**Keywords:** plant-insect interactions, phytohormones, larch adelgid, sink organs, cauline gall

## Abstract

The larch adelgid *Adelges laricis laricis* Vallot is a specialist insect parasite of *Picea koraiensis* (Korean spruce) and forms fish scale-like galls that damage the growth of the host plants. Our investigation reveals that both these galls and the fruits (cones) of *P. koraiensis* display lower concentrations of phytosynthetic pigments and accumulate anthocyanin cyanidin-3-*O*-glucoside and soluble sugars in the mature stages. Interestingly, high concentrations of 6-benzylaminopurine (BAP) both in the cauline gall tissues and in the larch adelgids themselves (4064.61 ± 167.83 and 3655.42 ± 210.29 ng/g FW, respectively), suggested that this vital phytohormone may be synthesized by the insects to control the development of gall tissues. These results indicate that the galls and cones are sink organs, and the development of gall tissues is possibly regulated by phytohormones in a way similar to that of the growth of cones. The concentrations of phytohormones related to growth [indole-3-acetic acid (IAA), cytokinins (CTK), and gibberellins (GAs)] and defense [salicylic acid (SA)], as well as SA-related phenolics [benzoic acid (BA) and *p*-hydroxybenzoic acid (*p*HBA)] in gall tissues were positively correlated with those in cones during the development stage. The levels of 1-aminocyclopropane-1-carboxylic acid (ACC) in the developmental stage of the cones correlates negatively with their concentrations in the gall tissues (*R* = −0.92, *p* < 0.001), suggesting that downregulation of ACC might be the reason why galls are not abscised after a year. Our results provide a new perspective on the potential mechanism of the development of cauline galls on *P. koraiensis*, which are regulated by phytohormones.

## Introduction

Herbivorous insects and plants are hugely important components of terrestrial communities and have coevolved over millions of years (Stahl et al., 2018). Damage caused by insect feeding on plants makes an important contribution to yield reductions across agricultural areas globally and is therefore responsible for considerable economic losses (Hilker and Fatouros, 2016). Insects are mobile and have evolved sophisticated and effective strategies to enable them to live on their host plants. Several different feeding habits are known and insects have evolved mouthparts that enable them to specialize in chewing, snipping, or sucking and have developed chemical-molecular crosstalk to cope with plant defenses (Howe and Jander, 2008). An example of a strategy evolved by an insect to counter plant host defenses is that the salivary secretions of certain herbivorous insect caterpillars contain the enzyme glucose oxidase, which counteract the production of defensive metabolites induced by the caterpillar feeding on the plant (Musser et al., 2002). Furthermore, the digestive proteases in the guts of phytophagous insects are able to counter plant defense proteins (Zhu-Salzman and Zeng, 2015). The complex chemical networks involved in the interactions between plants and insects resulting from thousands of years of evolution remain a hot topic in research.

One fascinating research area in the field of plant-insect interactions is the interaction between galling insects and plants. Insect galls are tumor-like organs on plant tissues, which are stimulated by the feeding of galling insects that can induce accelerated division of the host cells (Hirano et al., 2020). Galls can function as shelters and feeding sites for the insects inside and can develop in almost every plant organ, including roots, stems, leaves, flowers, fruits, and seeds. Galls have a negative impact on their host plants, slow plant growth, and can reduce plant height, leaf area, and production of inflorescences (Fay et al., 1996). However, these organs represent a close and sophisticated association between the galling insects and their host plants (Kutsukake et al., 2019). It is well-known that plant defenses against herbivory are triggered by insect-specific elicitors (Howe and Jander, 2008). Meanwhile, the insects counter these strategies by triggering multiple effective defense pathways, which can counter the plant defenses (Zhu-Salzman and Zeng, 2015). Certain insects secrete effectors, which are injected physically into host cells to control the plant defenses (Cambier et al., 2019). It is possible that these effectors potentially act as mediators responsible for the formation of arthropod-induced plant galls (Oliveira et al., 2016). Moreover, gall-inducing insects can manipulate host plant cells and tissues and control the formation of galls accompany chemicals. Therefore, understanding the mechanisms by which these insects can offset plant defenses and manipulate plants in other ways is a key to explain the complicated relationships between plants and galling insects.

Gall formation occurs when plant cell growth is accelerated following a stimulus caused by the feeding of galling insects (Stone and Schonrogge, 2003). Gall tissues usually absorb the photoassimilates and can manipulate the source-sink relationships in plants, allowing the formation and growth of galls resemblance to plant other organs (Dorchin et al., 2006; Oliveira et al., 2017). An example of insect alteration of plant source-sink relationships is found in the galling aphid, *Pemphigus betae*. Galls induced by these aphids use plant sources to maintain their own growth and development in the same way as other organs of plants do, including increasing size through cell hypertrophy and tissue hyperplasia (Larson and Whitham, 1997). A favorable microenvironment for the development of their galls is ensured by gall-inducing wasps, *Trichilogaster signiventris*, which can alter the photosynthetic capacity of the gall tissues to induce a resource sink (Castro et al., 2012). However, although the changes in source-sink relationships can lead to striking resemblances between the gall organs and other plant organs in the processes of growth and development, there are few studies focusing on this phenomenon and the mechanisms remain unclear.

Phytohormones are considered to be the pivotal regulators in the manipulation of plant tissues to enable the formation and growth of galls (Li et al., 2017; Body et al., 2019). These hormones may be secreted by the gall-inducing insects themselves or by the host plants (Straka et al., 2010; Tooker and Helms, 2014). In certain cases, galling insects have the ability to regulate plant hormones. Cytokinins (CTK, in most cases iP and *t*Z) secreted by gall-inducing insects and cause the galls to become strong photosynthate sinks, meaning that the insects inside are continually supplied with nutrients (Naseem et al., 2014; Takei et al., 2015). Gibberellins (GAs) and abscisic acid (ABA) play key roles in the regulation of gall formation and in insect-induced defensive responses (Li et al., 2017). Meanwhile, jasmonates (JAs) and salicylic acid (SA) are the key defensive phytohormones that mediate plant responses to galling herbivores during gall initiation and development (Eitle et al., 2019). The regulation of phytohormones is therefore of crucial importance in the formation and development of galls.

**Figure 1:**
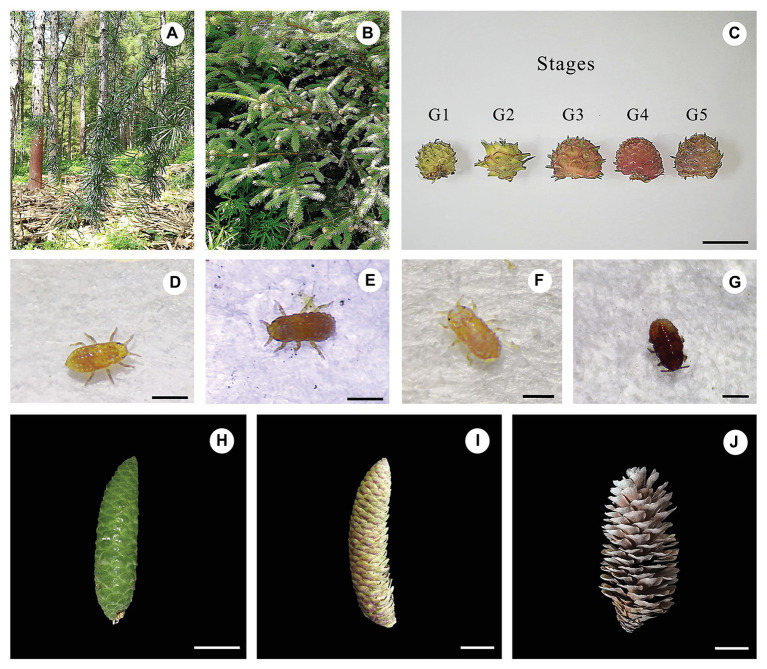
Field and microscope observations of the larch adelges *Adelges laricis laricis* Vallot and the cones of *Picea koraiensis*. Cauline galls induced by *A. laricis laricis* Vallot on *P. koraiensis*. **(A)** Field habit of *Larix kaempferi*, a secondary host of *A. laricis laricis*. **(B)** Field habit of *P. koraiensis*, a first host of *A. laricis laricis*. **(C)** Morphological characteristics of cauline galls caused by *A. laricis laricis* on *P. koraiensis* at different developmental stages of G1–G5. **(D–G)** *A. laricis laricis* Vallot isolated from the gall tissues of *P. koraiensis*. **(H–J)** *P. koraiensis* cones at different developmental stages of F1–F3. Scale bars = 2 cm for **(C)** and **(H–J)**; 0.25 mm for **(D)** and **(E)**; 0.4 mm for **(F)**; 0.35 mm for **(G)**.

The larch adelgid *Adelges laricis laricis* Vallot (Hemiptera, superfamily Phylloxeroidea, family Adelgidae) is polymorphic, commonly with a 2-year life cycle that involves different host plants (Yu et al., 1998). From field observations in Liaoning Province, China, *Picea koraiensis* (Korean spruce) was identified as a primary host plant for this adelgid. In the same area, the Japanese larch, *Larix kaempferi*, acts as a secondary host (Figure 1; Liu et al., 2011). Larch adelgids cause significant damage to young trees of *P. koraiensis*, affect the growth of stems and branches, and cause the formation of “fish scale” galls. On *L. kaempferi*, the larch adelgids suck the sap from the needles and shoots to produce large quantities of a white waxy secretion, resulting in dried and mildewed branches and seriously affecting tree growth (Figure 1; Yu et al., 1998; Liu et al., 2011). However, mechanism underlying gall formation in *P. koraiensis* is still poorly understood. In this study, we focus on exploring the dynamics of endogenous phytohormones in both galls and cones of *P. koraiensis* at different stages and investigate the roles of these hormones in the formation of galls. Our research contributes to a better understanding of the formation of gall tissues and supplies new insights into the potential mechanisms by which gall-inducing insects induce galls.

## Materials and Methods

### Plant Materials and Galling Insects

Galls and normal branches without galls were collected in July 2019 from *Picea koraiensis* trees and were growing in Wan dianzi town (E: 125°13', N: 41°96'), Fushun city, Liaoning province. Plants were identified by Professor Bo Qu, and voucher specimens (SYNUBL013060–SYNUBL013065) were deposited at the College of Bioscience and Biotechnology, Shenyang Agricultural University. Based on morphology, the development of these cauline galls can be divided into five stages (Figure 1). Galls at stage G1 were defined as those having gall tissues with white-green coloration. Stage G2 galls had deeper green coloration. Stages G1 and G2 together were defined as the “young” stage. The “mature” galls were divided into three stages of development, G3–G5, which are described by a gradual deepening of color from pale green to purple-black. The coloration of galls at stage G3 was turning from green to red. G4 galls were defined as completely red, and G5 galls were purple-black. The adelgid insects were almost totally absent from galls at stage G5. Cones were divided on the basis of morphology into three stages. Tender stage galls (green color, F1) were collected on July 1, 2019, mature stage galls (red, F2) were collected on September 1, 2019, and senescent stage galls (brown, F3) were collected on November 1, 2019.

Galls and cones were collected from five Korean spruce plants which were about 6 years old. Fifteen repeated gall and five repeated cones for each stage were collected. Galls were dissected in the laboratory to separate insects and gall tissues. Based on both morphological characters and molecular identification, the insects were identified as *Adelges laricis laricis* Vallot (larch adelgid) by Dr. Shouhui Sun, and voucher specimens (SYNUBC00045–SYNUBC00050) were deposited at the College of Bioscience and Biotechnology, Shenyang Agricultural University. Galls, adelgids, stems without galls, and cones were stored at −80°C for further analysis.

### Quantification of Phytohormones

A total of 26 SA-related phenolics and phytohormones were assessed, including 6-benzylaminopurine (BAP), 6-(γ,γ-dimethylallylamino) purine (iP), *trans*-zeatin (*t*Z), gibberellins (GAs, GA_1_, GA_3_, GA_4_, GA_7_, GA_9_, GA_12_, GA_19_, GA_24_, and GA_53_), indole-3-acetic acid (IAA), 3-indolepropionic acid (IPA), 1-aminocyclopropane-1-carboxylic acid (ACC), ABA, jasmonic acid (JA), salicylic acid (SA), methyl salicylate (MeSA), benzoic acid (BA), *p*-hydroxybenzoic acid (*p*HBA), *m*-hydroxybenzoic acid (*m*HBA), *p*-hydroxycinnamic acid (*p*HCA), *o*-hydroxycinnamic acid (*o*HCA), *m*-hydroxycinnamic acid (*m*HCA), and *trans*-cinnamic acid (*t*CA). The deuterated isotope-labeled compounds *d_2_*-GA_1_, *d_2_*-GA_4_, and *d_6_*-ABA were used as internal standards, which were purchased from Tokyo Chemical Industry Co., Ltd. (TCI, Tokyo, Japan). The concentrations of phytohormones and SA-related phenolics were quantified by external standard method. Phytohormones and SA-related phenolics were purchased from Tokyo Chemical Industry Co., Ltd. HPLC-grade solvents methanol (MeOH), formic acid, and acetonitrile were purchased from Merck. HLB and MCX solid-phase extraction cartridges were from Thermo (WondaSep HLB, MCX 60 mg/3 ml, 50EA/PKG).

The phytohormones with five biological replicates were extracted by following the previous descriptions with some slight modifications (Kojima et al., 2009). After removing larch adelgids from each gall chamber with a brush, the gall chambers (gall tissues) of each gall were prepared for a single sample. The cone and branches were chopped. After that, 500 mg fresh sample (gall tissues, branches, cones, or insects collected from the galls at stage G3) was homogenized using a precooled mortar pestle, and then ultrasonic-assisted extraction was conducted in 4 ml of extraction solution (methanol:water:formic acid = 15:4:1) for 45 min. After centrifugation at 12,000 rpm for 10 min, the supernatant was collected, the residue was ultrasonically reextracted and centrifuged by following the above method, and the extraction supernatants from both steps were combined. Samples and solutions were kept at 4°C throughout the extractions. HLB and MCX columns were preactivated with 2 ml methanol and 2 ml of 1 M formic acid. Every 2 ml of the supernatants loaded onto an HLB column and successively washed with 1 ml of the extraction solution. The eluates and washing solution were collected together and concentrated at 40°C under a rotary evaporator to get about 1 ml solution. This solution was passed through a MCX column eluting with 0.5 ml of 1 M formic acid and 2 ml methanol. And the methanol fraction was concentrated and redissolved in 0.2 ml of methanol. Then, the redissolved solutions were filtered through a 0.22 μm filter and transferred to 2 ml LC-MS glass bottles for UPLC-MS/MS analysis. The concentrations of GAs, IAA, IPA, ABA, JA, SA, MeSA, BA, *p*HBA, *m*HBA, *p*HCA, *o*HCA, *m*HCA, and *t*CA were analyzed. For analyses of BAP, iP, and *t*Z, the MCX column was sequentially eluted with 500 ml 0.35 M ammonia and 2 ml 0.35 M ammonia in 60% (v/v) methanol. The 0.35 M ammonia in 60% (v/v) methanol fraction was concentrated and redissolved in 0.2 ml of methanol for analysis of BAP, iP, and *t*Z using UPLC-MS/MS.

UPLC-MS/MS analyses were conducted using an UPLC-MS/MS system (Shimadzu LCMS-8050) with a Shim-pack GIST C_18_ (2 μm, 2.1 × 100 mm). The mobile phase consisted of 0.1% formic acid (A) and acetonitrile (B). The gradient elution was programed as follows: 0–2 min, 20–30% B; 2–8 min, 30% B; 8–12 min, 30–95% B; 12–14 min, 95% B; and 14–16 min, 95–20% B. A flow rate of 0.4 ml/min was used, and the injection volume was 10 μl. The column temperature was maintained at 40°C. The operating conditions of the electrospray ionization source (ESI) were as follows: gas flow 3 L/min; heating gas 10 L/min; dry gas flow 10 L/min; interface temperature, 300°C; and heating block temperature, 450°C. All the compounds were monitored using the multi reaction monitoring (MRM) mode, and the specific MRM parameters for each compound are given in Supplementary Table S1.

ACC was extracted as described previously (Ziegler et al., 2014). In short, the extract solutions and loading onto HLB columns were consistent with the methods of other phytohormones without MCX columns. The eluates of HLB column were evaporated to dryness at 37°C in a rotary evaporator. A total of 40 μl reaction buffer (anhydrous ethanol:water:triethylamine:PITC = 2:1:1:1) was added to the rotary evaporator bottle. After redissolving, the samples were left at room temperature for 20 min to react and were then evaporated to dryness as before. A further 50 μl 40% acetic acid was added and allowed to react at 90°C for 1 h. Then the reaction solution was evaporated and the residue redissolved in 500 μl methanol. The ACC was quantitatively analyzed using an UPLC-MS/MS system (Shimadzu LCMS-8050) with a Shim-pack GIST C_18_ (2 μm, 2.1 × 100 mm). The mobile phases were 0.1% formic acid (A) and acetonitrile (B). The gradient elution was conducted as follows: 0–13 min, 5–95% B. The flow rate was 0.4 ml/min and the volume of injection was 10 μl. The column temperature was maintained at 35°C. The electrospray ionization source (ESI) with multi reaction monitoring (MRM) mode was applied in MS detection (Supplementary Table S2).

### Qualitative Analysis of Anthocyanins in Gall Tissues and Cones Using HPLC-DAD

After the insects inside the galls were removed, about 500 mg of the gall tissues (G3) or cones (F2) was ground into powder in liquid nitrogen and then suspended with 5 ml 80% methanol/water (*v*/*v*) with 2‰ formic acid in an ultrasonic bath for 45 min. The suspension was then centrifuged at 12,000 rpm for 5 min. After centrifugation, the supernatant was concentrated and evaporated to dryness. The supernatant was then suspended in 1 ml methanol and then analyzed using UPLC-MS/MS (UPLC-MS/MS 8050, Shimadzu Scientific Instruments, Inc., Tokyo, Japan) and HPLC-DAD (Agilent 1260). Samples were injected using an autosampler (SIL-30 AC; Shimadzu) into a mobile phase comprising acetonitrile (B) and 2% formic acid in water (D; 0–10 min: isocratic 15% B; 10–13 min, linear 15–95% B, 13–14 min, isocratic 95% B). The flow rate was 0.2 ml/min with injection volume of 10 μl. Cyanidin-3-*O*-glucoside was dissolved in methanol at 100 μg/ml and then analyzed by HPLC-DAD. At a flow rate of 1 ml/min, 10 μl of the sample was then injected into an Eclipse XDB-C_18_ column (5 μm, 4.6 × 250 mm), with the column temperature maintained at 30°C. The eluent was monitored at 200–600 nm. Mobile phases comprising (B) methanol and (D) 2% formic acid were used (0–30 min: linear 5–95% of B, 30–40 min, isocratic 95% of B).

### Determination of the Concentrations of Photosynthetic Pigments in Gall Tissues and Cones

Pigments were extracted from fresh control branches, gall tissues (G1–G5), and cones (F1–F3) tissues. Each sample was treated with five biological replicates. After removing the galling insects, the fresh gall tissue or cone (200 mg) samples were suspended in 5 ml 80% acetone/water (*v*/*v*) and then ground with a pestle and mortar until all the tissues with visible pigmentation had disappeared. A 3 ml aliquot of the extract was transferred to a cuvette for the determination of chlorophyll absorption using a spectrophotometer at 470, 646, and 663 nm. Absorption measurements were used to quantify the concentrations of chlorophylls *a*, and *b*, and of total carotenoids, based on the equations reported in previous publications (Wellburn, 1994).

### Determination of the Concentrations of Soluble Sugars in Gall Tissues and Cones

Samples were prepared with five biological replicates as described above for the analyses of glucose, fructose, and sucrose (Guimarães et al., 2009). Briefly, 1 g of fresh gall tissues or cones were weighed and dried to a constant weight (105°C, 5 min and 80°C, 30 min). Dry gall tissues and cones were homogenized using a mortar pestle and transferred to the test tube. Then, samples were extracted in 80% aqueous ethanol for 40 min in the water bath at 80°C. The supernatant was collected, and the residue was reextracted two times under the same conditions. The supernatants from previous steps were combined. Samples with extraction solutions were shaken every 10 min throughout extractions. A total of 20 mg activated carbon was added and decolorized by shaking at 80°C for 30 min (shaking per 5 min). Further filtration with a funnel to rotary evaporator bottle was conducted and concentrated at 40°C under a reduced pressure and redissolved in 1 ml ultrapure water. Then, the redissolved solutions were filtered through a 0.22 μm filter for further HPLC (Agilent 1260) coupled to a refraction index detector (RID) analysis. The mobile phase consisted of the following linear gradients (flow rate, 1 ml/min; injection volume, 10 μl): 0–30 min, 20% A, 80% C (A was ultrapure water and C was acetonitrile). The column was Innovation NH_2_ (Chrom-ma Trix Bio-Technology; 5 μm, 4.6 × 250 mm) and column temperature maintained at 35°C. Standard solutions of glucose, fructose, and sucrose were prepared in the range of 0.625–10 mg/ml. Furthermore, the calibration curves of glucose, fructose, and sucrose were *y* = 1E-05x−0.065 (*t*_R_, 9.7 min; *R* ^2^ = 0.999), *y* = 1E-05x−0.027 (*t*_R_, 8.4 min; *R* ^2^ = 0.996), and *y* = 2E-05x−0.017 (*t*_R_, 13.8 min; *R* ^2^ = 0.991), respectively.

### Statistical Analysis

The statistical analysis and graphics were conducted using ggstatplot (v 0.1.4). If the data followed a normal distribution, an independent-samples’ *t*-test was used for comparison between the two groups, and the comparison among three or more groups was performed using one-way ANOVA with Tukey’s test. If results of the *t*-test were significant and with the data were not normally distributed, a Mann-Whitney nonparametric test was used to compare two groups of data. Differences were considered to be statistically significant if *p* ≤ 0.05. Corrplot and corrgram were used for the calculation of the correlation coefficient and for visualization. The R program (www.r-project.org) was used for statistical analysis and calculations.

## Results

### Galls and Cones on *Picea koraiensis* Are Sink Organs

Based on morphological characters and DNA sequence analysis of DNA barcodes (*COI*), the gall insects in the stems of *Picea koraiensis* (Korean spruce) were identified as *Adelges laricis laricis* Vallot (Yu et al., 1998; Žurovcová et al., 2010).

Cones of Korean spruce are about 70–90 mm long and have a scaly surface. Each scale protects a seed. We split the development of the cones roughly into three stages: tender (F1), mature (F2), and senescent (F3). Galls caused by larch adelgids on *P. koraiensis* are about 15–20 mm both in length and width and also have a fish-scale like surface with strawberry shaped. The surface of the galls has a regular arrangement of cone-scale-like protuberances. Each protuberance has a split line and bundles of needles of varying lengths. The line of dehiscence appears white, orange-red, and finally pink before splitting. Each protuberance is in fact a highly organized and differentiated gall chamber, inhabited by the *A. laricis laricis* Vallot insects. Gall chambers are independent of each other and are irregularly arranged. Thus, from morphology, the larch adelgid galls resemble the cones of *P. koraiensis* (Figure 1).

Concentrations of glucose, fructose, and sucrose in fresh gall tissues and cones analyzed using HPLC-RID. The concentrations of these sugars increased throughout gall development. The higher concentrations of glucose, fructose, and sucrose in mature stage of gall tissues were observed than those of normal branches. Similarly, the higher concentrations of glucose and fructose exhibited in fresh mature cones (F2), with values of 1.56 ± 0.02 and 3.31 ± 0.22 mg/g FW, respectively (Supplementary Figure S1).

**Figure 2:**
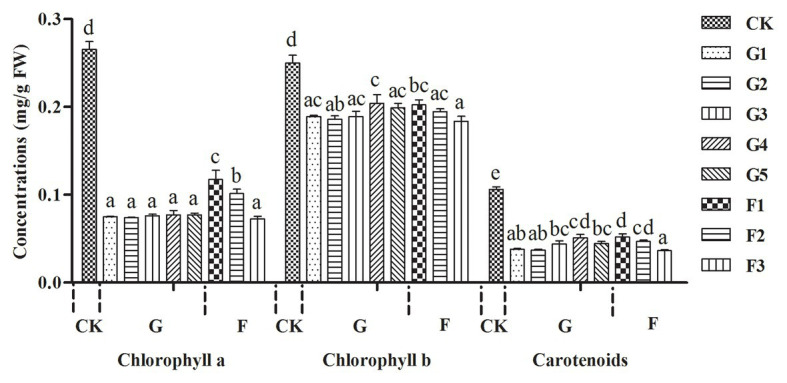
Variations in concentration of photosynthetic pigments in galls tissues and the cones of *Picea koraiensis*. *CK* = normal branches. Data are presented in milligrams per gram of fresh weight (mg/g FW). G1–G5 represent the different developmental stages of gall tissues and F1–F3 represent different developmental stages of cones. G: gall tissues; F: cones. Mean differences were compared using one-way ANOVA with Tukey’s test. The different small letters (a, b, c, and d) represent significant difference at 0.05 level. These results shown represent the average ± SD.

**Figure 3:**
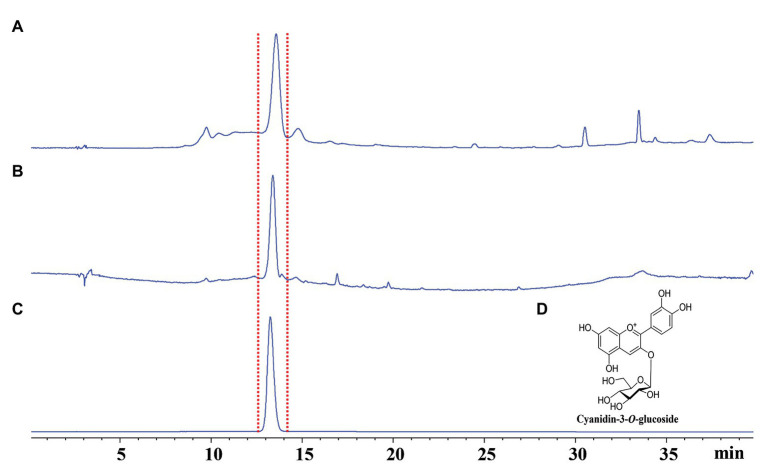
Qualitative analyses of fresh gall tissues at stage G4 and cones of *Picea koraiensis* at stage F2 using HPLC-DAD at 520 nm. **(A)** HPLC analysis of gall tissues at stage G4; **(B)** HPLC analysis of cones of *Picea koraiensis* at stage F2; **(C)** HPLC analysis of cyanidin-3-*O*-glucoside (100 μg/ml); and **(D)** Chemical structures of cyanidin-3-*O*-rutinoside.

Concentrations of chlorophylls *a* and *b* and carotenoids were higher in normal branches than those in fresh gall tissues and cones, but there were no significant differences in the concentrations of these photosynthetic pigments between gall tissues and cones (Figure 2). The red coloration of the galls and cones of *P. koraiensis* is mainly due to the accumulation of anthocyanin pigments using HPLC-DAD analysis. Through the comparison with the standard, the main coloration in mature stage of gall tissues and cones was identified as cyanidin-3-*O*-glucoside (Figure 3). Thus, galls and cones are therefore both sink organs on the aerial parts of Korean spruce plants.

### Growth Related Phytohormones in the Gall Tissues and Cones Throughout the Developmental Stages

**Figure 4:**
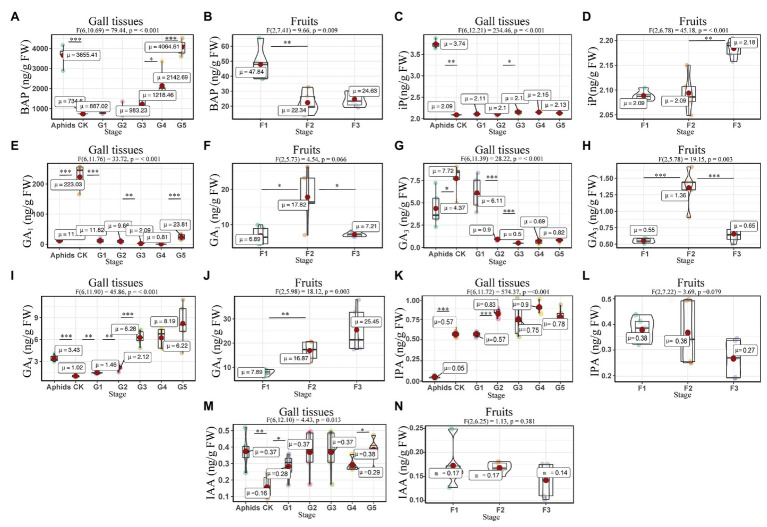
Quantitative analysis of growth related phytohormones in the larch adelgids, gall tissues, and cones (fruits) of *P. koraiensis*. Quantitative analysis of 6-benzylaminopurine (BAP) in larch adelgids, gall tissues, normal branches **(A)**, and cones **(B)**. The level of 6-(γ,γ-dimethylallylamino) purine (iP) in gall tissues and normal branches **(C)** and cones **(D)**. The level of GA_1_ in larch adelgids, gall tissues, and normal branches **(E)** and cones **(F)**. Quantitative analyses of GA_3_ and GA_4_ in larch adelgids, gall tissues, and normal branches (**G** and **I**), and cones (**H** and **J**), respectively. The Levels of IPA and IAA in larch adelgids, gall tissues, and normal branches (**K** and **M**), and cones (**L** and **N**), respectively. *CK* = normal branches. Data are presented in nanograms per gram of fresh weight (ng/g FW), and boxplots display the minimum, first quartile, median, third quartile, and maximum for each stage in the given tissues. G1–G5 represent the different developmental stages of gall tissues and F1–F3 represent different developmental stages of cones. Red point = mean value. Horizontal black line inside the box = median value. The shape of the boxplot indicates the distribution of results. Mean differences were compared using *t*-tests. ^*^Denotes that the value of *p* is less than 0.05. ^**^Denotes that the value of *p* is less than 0.01. ^***^Denotes that the value of *p* is less than 0.001. These results shown represent the average ± SD and performed one-way ANOVA test among three or more groups. *ω_p_* ^2^ = partial omega squared.

The concentration of 6-benzylaminopurine (BAP) increased with the development of the gall tissues, reaching the value of 4064.61 ± 167.83 ng/g FW in the last stage. Furthermore, the concentration of BAP in the larch adelgids was 3655.42 ± 210.29 ng/g FW, which is four times higher than BAP concentrations in either normal branches or gall tissues at the related stage (G3, Figures 4A,B). Another cytokinin, 6-(γ,γ-dimethylallylamino) purine (iP), was found in the larch adelgids, but its concentration did not appear to be correlated with the development of gall tissues or the cones in Korean spruce (Figures 4C,D). The concentrations of auxins (IAA and IPA) and gibberellins (GA_1_, GA_3_, and GA_4_) were low in all treatments, except that there were higher concentrations of GA_1_ in the normal branches of *P. koraiensis*, with concentrations of 223.03 ± 18.68 ng/g FW. Concentrations of GA_4_ in gall tissues increased throughout development (Figures 4E–N). Thus, it is possible that the abnormal growth of the gall tissues might be stimulated by BAP synthesized by the larch adelgid insects.

**Figure 5:**
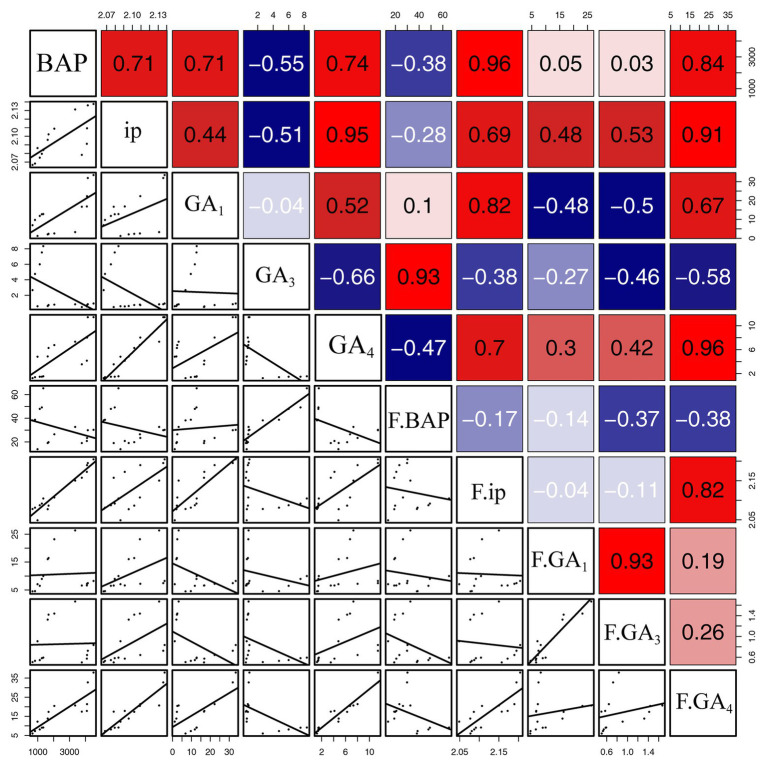
Correlation of growth-related phytohormones between gall tissues and cones. The mean differences were compared using *t*-test (*p* < 0.05). BAP, iP, GA_1_, GA_3_, and GA_4_ represent phytohormone levels in gall tissues; F.BAP, F.iP, F.GA_1_, F.GA_3_, and F.GA_4_ represent phytohormone levels in cones. Red and blue squares represent positive and negative correlations, respectively.

Correlation analysis suggested that the concentrations of BAP were significantly and positively related to concentrations of iP (*R* = 0.71, *p* < 0.01), GA_1_ (*R* = 0.71, *p* < 0.01), and GA_4_ (*R* = 0.74, *p* < 0.01) in gall tissues, and furthermore that concentrations of iP were significantly and positively correlated with concentrations of GA_4_ (*R* = 0.95, *p* < 0.001) in gall tissues. Moreover, there was a positive correlation between concentrations of GA_1_ and GA_4_ in gall tissues (*R* = 0.52, *p* < 0.05). In addition, there were negative correlations between concentrations of GA_3_ and the other four growth related phytohormones in gall tissues, among which there was a significant negative correlation of GA_3_ with GA_4_ (*R* = −0.66, *p* < 0.01). There were negative correlations between concentrations of BAP and the other four growth related phytohormones during cone development. Concentrations of iP and GA_4_ (*R* = 0.82, *p* < 0.001) were significantly and positively correlated. Concentrations of GA_1_ were positively correlated with GA_3_ (*R* = 0.93, *p* < 0.001) in cones. Furthermore, there was a positive, although not significant, correlation between GA_3_ and GA_4_ (Figure 5).

### Defense Related Phytohormones in the Gall Tissues and Cones During the Developmental Stages

**Figure 6:**
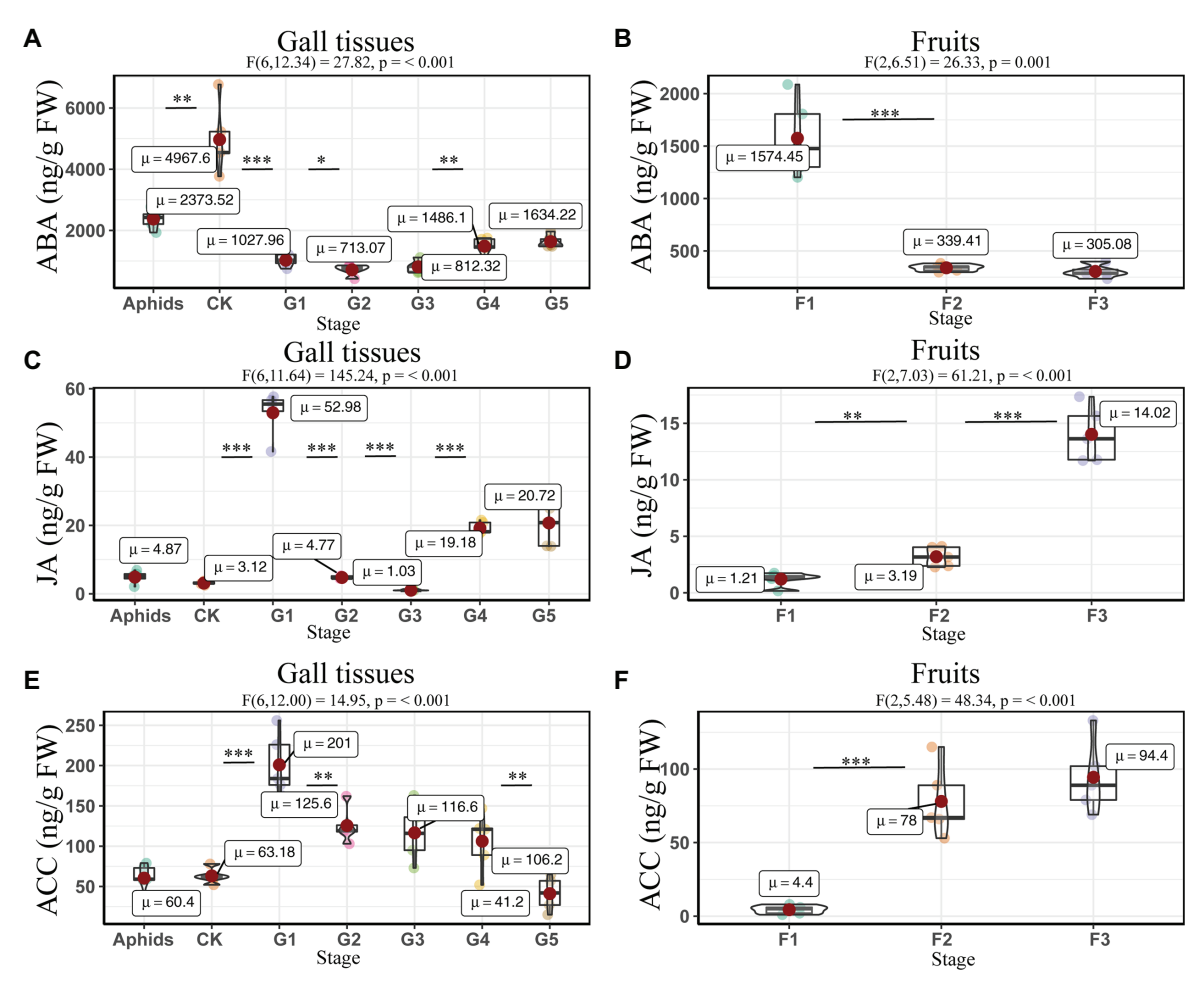
Quantitative analysis of defense related phytohormones concentrations in larch adelgids, gall tissues, and cones of *P. koraiensis*. The abscisic acid (ABA), jasmonic acid (JA), and 1-aminocyclopropane-1-carboxylic acid (ACC) concentrations in gall tissues and normal branches **(A,C,E)** and cones **(B,D,F)**, respectively. *CK* = normal branches without gall. Data are presented in nanograms per gram of fresh weight (ng/g FW), and boxplots display the minimum, first quartile, median, third quartile, and maximum for each stage in the given tissues. Red point = mean value and horizontal black line inside the box = median value. The shape of the boxplots indicates distribution of results. The mean differences were compared using *t*-tests. ^*^Indicates that the value of *p* is less than 0.05. ^**^Indicates that the value of *p* is less than 0.01. ^***^Indicates that the value of *p* is less than 0.001. These results shown are the average ± SD and performed one-way ANOVA test among three or more groups. *ω_p_* ^2^ = partial omega squared.

The concentrations of ABA and JA were affected by the developmental stage in both the gall tissues (ABA: F6, 12.13 = 27.82, *P* = <0.001; JA: F6, 11.64 = 145.24, *P* = <0.001) and Korean spruce cones. Similar expression patterns of ABA and JA in the developmental stages of the gall tissues were observed, in which the concentrations decreased throughout stages G2 and G3 and then increased again in stages G4 and G5 compared to stage G1 (Figures 6A,C). The normal gall-free branches of Korean spruce and cones at stage F1 showed the highest ABA concentrations, with values of 4967.60 ± 505.83 and 1574.46 ± 164.34 ng/g FW, respectively (Figures 6A,B). Concentrations of JA and ACC increased throughout cone development (Figures 6D,F). However, concentrations of ACC decreased throughout the developmental stages of the galls (Figure 6E).

**Figure 7:**
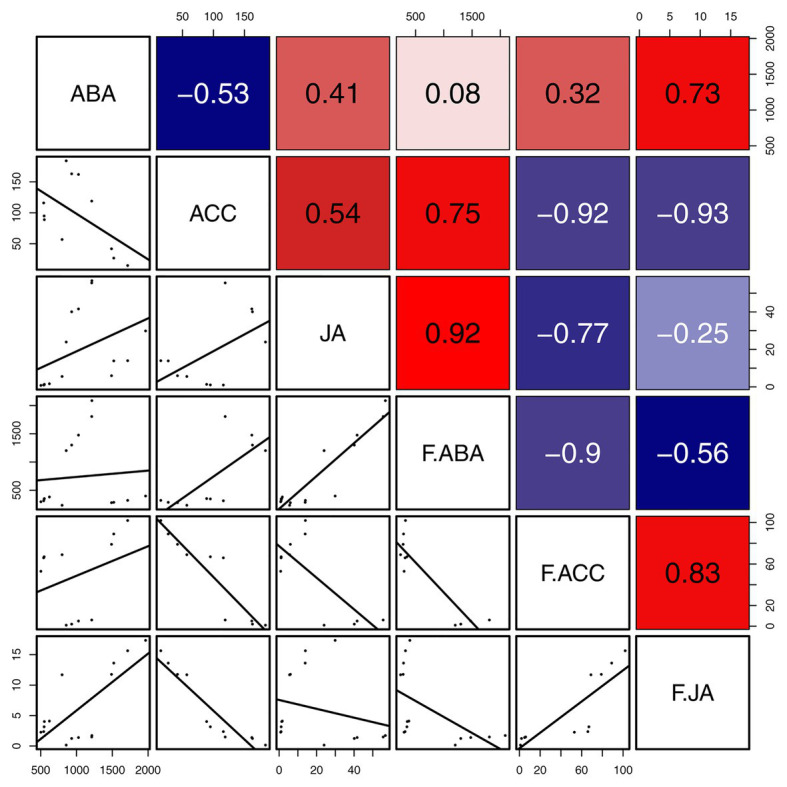
Correlation of defense related phytohormones between gall tissues and cones. Mean differences were compared using *t*-tests (*p* < 0.05). Red and blue boxes represent positive and negative correlations, respectively.

The correlation analyses suggested that the concentrations of JA were positively correlated with those of ACC and ABA in gall tissues. During cone development, the concentrations of JA were significantly and negatively correlated with the ABA concentrations (*R* = -0.56, *p* < 0.05), and the concentrations of ABA were significantly and negatively correlated with those of ACC (*R* = −0.90, *p* < 0.001). In addition, there was a significant positive correlation between concentrations of JA and ACC (*R* = 0.83, *p* < 0.01). The concentrations of ACC and JA in the cones were negatively correlated with the corresponding phytohormones in the galls; however, only the concentrations of ACC showed a significant negative correlation between cones and galls (*R* = −0.92, *p* < 0.001). These results suggest that ACC cooperates with JA and ABA in the development of both galls and cones. Moreover, galls that do not abscize and can account for the downregulated ACC result in any current year. Upregulated ACC might therefore be triggered the abscission of the cones (Figure 7).

### SA and SA-Related Phenolics in Gall Tissues and Cones Throughout the Developmental Stages

**Figure 8:**
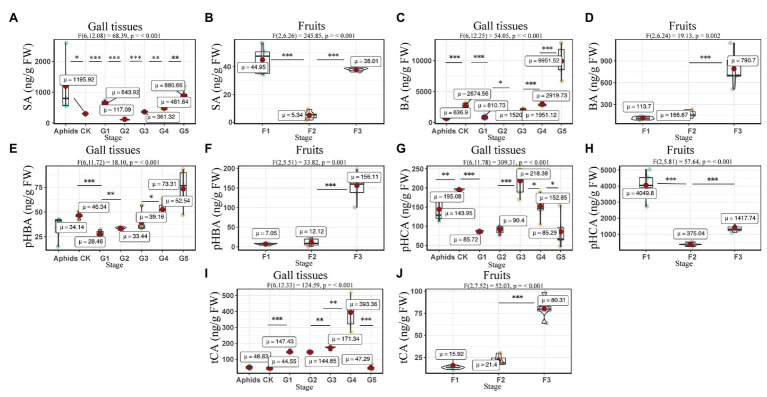
Quantitative analysis of concentrations of SA and SA-related phenolics in larch adelgids, gall tissues, and *P. koraiensis* cones. Quantitative analysis of salicylic acid (SA), benzoic acid (BA), *p*-hydroxybenzoic acid (*p*HBA), *p*-hydroxycinnamic acid (*p*HCA), and *trans*-cinnamic acid (*t*CA) in gall tissues and normal branches **(A,C,E,G,I)** and cones **(B,D,F,H,J),** respectively. *CK* = normal branches. Data are presented in nanograms per gram of fresh weight (ng/g FW), and boxplots display the minimum, first quartile, median, third quartile, and maximum for each stage in the given tissues. Red point = mean value and horizontal black line inside the box = median value. Shape of boxplots indicates distribution of results. The mean differences were compared using *t*-tests. ^*^indicates that the value of *p* is less than 0.05. ^**^Indicates that the value of *p* is less than 0.01. ^***^Indicates that the value of *p* is less than 0.001. The results shown are an average ± SD and performed one-way ANOVA test among three or more groups. *ω_p_* ^2^ = partial omega squared.

**Scheme 1:**
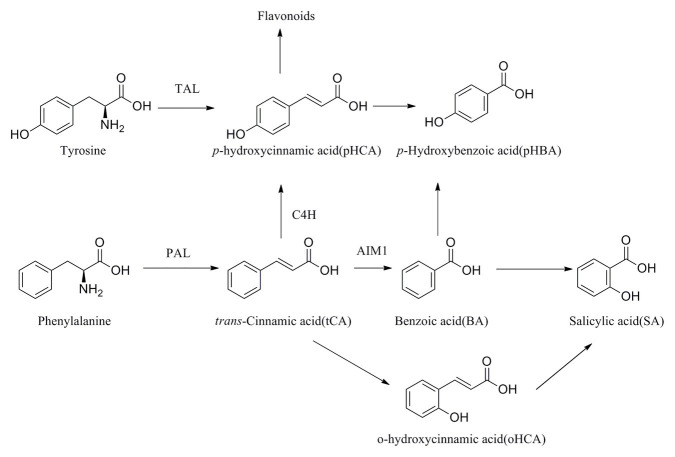
Plausible biosynthesis pathway of SA-related phenolics.

The concentrations of BA and *p*HBA were affected by developmental stage (gall tissues: BA: F6, 12.25 = 54.05, *p* = <0.001; *p*HBA: F6, 11.72 = 18.10, *p* = <0.001; cones: BA: F2, 6.24 = 19.13, *p* = 0.002; *p*HBA: F2, 5.51 = 33.82, *p* = 0.001). The BA in gall tissues exhibited the highest concentrations in the last stage of development, with a value of 9951.52 ± 1108.53 ng/g FW, which was four times that in the control branches and 13 times that in the mature cones (Figure 8). The highest concentrations of SA in the gall tissues were also observed in the last stage of gall development, with a value of 880.66 ± 98.05 ng/g FW, which was three times that in the control branches and 23 times that in mature cones. Interestingly, high concentrations of SA were also found in the larch adelgids, with values of 1195.92 ± 386.55 ng/g FW, indicating that the larch adelgids have the ability to store or transport SA. Compared with concentrations in gall tissues at other developmental stages, the upregulated concentrations of *t*CA and *p*HCA at stages G3 and G4 (Figure 8) were correlated with the concentration of anthocyanins at those stages, suggesting that *t*CA and *p*HCA are the key precursors in the biosynthesis of anthocyanins (Scheme 1). The concentrations of *t*CA, BA, and *p*HBA in the Korean spruce cones also increased with developmental stage.

**Figure 9:**
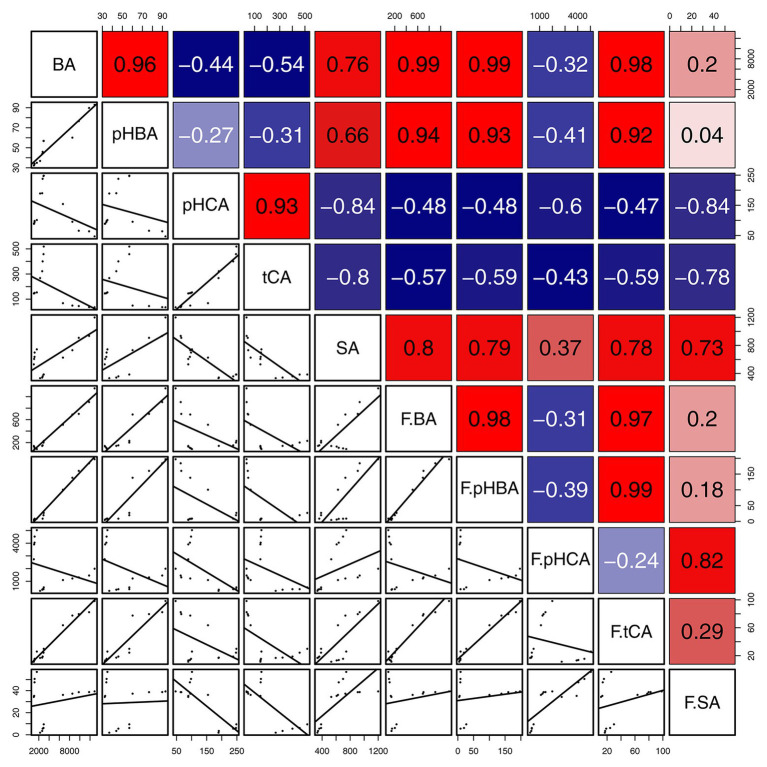
Correlation of SA and SA-related phenolics between gall tissues and cones. The mean differences were compared using *t*-tests (*p* < 0.05). BA, *p*HBA, *p*HCA, *t*CA, and SA represent the levels of phytohormones in gall tissues; F.BA, F.*p*HBA, F.*p*HCA, F.*t*CA, and F.SA represent the phytohormone levels in cones. Red and blue boxes represent positive and negative correlations, respectively.

The concentrations of BA in gall tissues were positively correlated with those of *p*HBA (*R* = 0.96, *p* < 0.001) and SA (*R* = 0.76, *p* < 0.01), and we also observed a significant positive correlation between the concentrations of *p*HBA and SA (*R* = 0.66, *p* < 0.05) in gall tissues. These results suggest that BA might be the precursor of *p*HBA and SA (Scheme 1). In addition, concentrations of *p*HCA in gall tissues were significantly and positively correlated with those of *t*CA (*R* = 0.93, *p* < 0.001) throughout the developmental stages. Similarly, throughout cone development, concentrations of BA were significantly and positively correlated with those of *p*HBA (*R* = 0.98, *p* < 0.001), *t*CA (*R* = 0.97, *p* < 0.001) and were also positively correlated with SA concentrations, although this relationship was not significant. In addition, there was a significant positive correlation between *p*HBA and *t*CA (*R* = 0.99, *p* < 0.001) in cones. The correlative patterns of *p*HBA, BA, and SA in cones were consistent with those in insect-induced galls. However, concentrations of *p*HCA and *t*CA in gall tissues were strongly and negatively correlated with those in cones. With the exception of *p*HCA and *t*CA, our results demonstrate that changes in the concentrations of SA-related phenolics during gall development are consistent with those during cone development (Figure 9).

## Discussion

### Both Galls and Cones Are Sink Organs

Together with flowers, buds, stems, and roots, fruits are important natural plant sink organs that absorb photoassimilates from adjacent tissues to maintain their own normal growth and development (Dorchin et al., 2006; Samsone et al., 2011). The behavior of galls as assimilate sinks has been widely studied, and they compete with natural plant sink organs for the nutrient supply that supports their formation and growth (Yang et al., 2007; Huang et al., 2011, 2015). Galls can affect the carbon partitioning mechanisms within their host plant because they can alter the resource balance between source and sink tissues (Dorchin et al., 2006). The high level of sugars in the gall tissues evidenced that they can be sink organs on the stem of *P. koraiensis*. Consequently, galls have a negative impact on the growth and development of the host plant, leading to reductions in the production of flowers, fruits, seeds, or biomass (Oliveira et al., 2017). There is therefore a competitive relationship between the gall and fruit sinks. Based on close source-sink relationships, the developmental processes of galls and fruit are similar. However, the mechanisms of this phenomenon are not fully understood.

### 6-Benzylaminopurine Stimulates the Development of the Gall Tissues

Endogenous 6-benzylaminopurine (BAP) and its derivatives have been detected in several plant species. Furthermore, BAP has been detected in many different parts of both the coconut palm (*Cocos nucifera* L.) and the Baikal skullcap (*Scutellaria baicalensis* Georgi; Nandi et al., 1989; Sáenz et al., 2003; Chernyad’ev, 2009). In our study, we found that the concentrations of the cytokinin BAP in the gall-inducing larch adelgids were significantly higher than those in either normal branches without galls or gall tissues at stages G1–G3. These results suggest that BAP is synthesized by the insects themselves to promote the formation of galls. Exogenous treatment with BAP will delay the leaf abscission of soybean (Kuang et al., 1992) indicated high level of BAP in last stage of gall tissues related to anti-abscission of the gall after a year. However, only the concentrations of BAP showed a little negative correlation between cones and gall tissues (*R* = −0.38; Figure 5). Thus, the anti-abscission of the *P. koraiensis* cones may be unrelated to the higher concentrations of BAP. Moreover, higher concentrations of iP and *t*Z have been found in gall-inducing insects (*Pontania* sp., *Eurosta solidaginis, Stenopsylla nigricornis*, and others) than in plant tissues (Mapes and Davies, 2001; Yamaguchi et al., 2012; Kai et al., 2017). It is therefore inferred that iP and *t*Z synthesized by the galling insects might manipulate host plants to facilitate the expansion of gall tissues (Mapes and Davies, 2001). However, in our experiments, the concentrations of iP and *t*Z in larch adelgids and in the gall tissues of *P. koraiensis* were relatively low, suggesting that they were not the key substances responsible for *A. laricis laricis* gall expansion on *P. koraiensis*.

### The Accumulation of Anthocyanin Is Correlated to Sink Function of Gall Tissues

Anthocyanin acting as one of main coloring matters widely distributes in sink organs of plants (Wang et al., 2020). The red color observed in insect gall tissues is mainly caused by the presence of anthocyanins in those tissues (Gerchman et al., 2013). This has led to the possible connection between gall development and anthocyanins. Some researchers have found that this occur is due to the upregulation of the phenylpropanoid pathways by cytokinins in response to galler attack, which leads to anthocyanin accumulation in the galls (Connor et al., 2012). However, the upregulated phenylpropanoids may damage the photosynthetic system, which led to these tissues sensitive to UV radiation (Zhou et al., 2020). In order to reduce the damage of UV radiation, plants accumulate anthocyanins in these tissues (Connor et al., 2012). Moreover, the upregulation of anthocyanins may protect the galling aphids against UV radiation in peach species (Zhou et al., 2020). Totally, the accumulation of anthocyanins in gall tissues is correlated to their sink function.

### The Lack of Abscission of Galls Is Related to the Concentrations of 1-Aminocyclopropane-1-Carboxylic Acid

Fruit abscission is closely related to the regulation of plant hormones (Iqbal et al., 2017). The endogenous hormone ethylene has a major role in the process of fruit abscission, in which there is also crosstalk with the other hormones ABA, GAs, auxins, and CTK (Iqbal et al., 2017). The concentration of ACC is known to be positively correlated with to the fruit development and abscission (Samsone et al., 2012; McAtee et al., 2013). However, our observations of ACC in the *P. koraiensis* galls suggest that the concentrations of this phytohormone decrease with gall developmental stage. Furthermore, the galls on the stem of *P. koraiensis* do not abscise the year they form and can remain on the tree for at least 2 years. Hence, the lack of abscission of the *P. koraiensis* galls may related to the low concentrations of ACC.

### The Functions of SA and SA-Related Phenolic Acids in Galls and Cones of Korean Spruce

SA and SA-related phenolic acids are broadly distributed throughout plant tissues and are closely related to the growth of plant (Eitle et al., 2019). The biosynthesis of these phenolic acids occurs *via* both the phenylalanine and the tyrosine pathway (Monica et al., 2016). In the phenylalanine pathway, phenylalanine is initially processed to *t*CA by phenylalanine ammonia lyase (PAL) and then to *p*HCA by cinnamate 4-hydroxylase (C4H), which leads on to the flavonoid pathway (Scheme 1). Different biosynthetic pathways are then employed to convert *t*CA to either *p*HBA or SA.

Phenolic acids are also important in plant responses to biotic and abiotic stresses, particularly against fungal infections (Firn, 1989). With the developmental stage of gall tissues, there is an increased risk of fungal infection with development of galls, because of the dehiscence (Amborabé et al., 2002). Therefore, it is speculated the higher concentrations of the phenolics BA and *p*HBA observed in galls may be related to their role in defense against fungal attack. In addition, the phenolics *t*CA and *p*HCA are vital precursors of anthocyanin synthesis (Hilker and Fatouros, 2016). In *P. koraiensis* gall tissues, *t*CA and *p*HCA concentrations reached the highest levels at stages G4 and G3, respectively, where the gall tissues turn red, probably due to the accumulations of anthocyanins. This is similar to the accumulation of *t*CA throughout cone development, and the concentrations of *p*HCA in cone tissues were very high, which may also be associated with accumulation of anthocyanins and changes in color. Therefore, the role of anthocyanins in *P. koraiensis* galls and cones may be very similar.

## Conclusion

All the present results suggest that phytohormones can act as the key regulators in the growth and development of both *P. koraiensis* galls and cones. This agrees with the results from the current study, which demonstrated that both galls and cones use a source-sink relationship for their own growth and development, resulting in similar appearance and morphology. In particular, BAP secreted by larch adelgids facilitates the formation of *P. koraiensis* galls. Our study provides further insights into gall formation and focuses on the potential mechanism by which the galling insects can induce gall tissues.

## Data Availability Statement

The raw data supporting the conclusions of this article will be made available by the authors, without undue reservation.

## Author Contributions

MJ, WZ, QL, JL, and SL designed the research and performed the experiments. MJ, WZ, JH, QL, and BQ analyzed the data. SL and MJ wrote the paper and conceived the project. All authors contributed to the article and approved the submitted version.

## Conflict of Interest

The authors declare that the research was conducted in the absence of any commercial or financial relationships that could be construed as a potential conflict of interest.

## References

[ref1] Amborabé B. E. Fleurat-Lessard P. Chollet J. -F. Roblin G. (2002). Antifungal effects of salicylic acid and other benzoic acid derivatives towards *Eutypa lata*: structure–activity relationship. Plant Physiol. Biochem. 40, 1051–1060. 10.1016/S0981-9428(02)01470-5

[ref2] Body M. J. A. Zinkgraf M. S. Whitham T. G. Lin C. H. Richardson R. A. Appel H. M.. (2019). Heritable phytohormone profiles of poplar genotypes vary in resistance to a galling aphid. Mol. Plant-Microbe Interact. 32, 654–672. 10.1094/MPMI-11-18-0301-R, PMID: 30520677

[ref3] Cambier S. Ginis O. Moreau S. J. M. Gayral P. Drezen J. -M. (2019). Gall wasp transcriptomes unravel potential effectors involved in molecular dialogues with oak and rose. Front. Physiol. 10:926. 10.3389/fphys.2019.00926, PMID: 31396099 PMC6667641

[ref4] Castro A. C. Oliveira D. C. Moreira A. S. F. P. Lemos-Filho J. P. Isaias R. M. S. (2012). Source–sink relationship and photosynthesis in the horn-shaped gall and its host plant *Copaifera langsdorffii* Desf. (Fabaceae). S. Afr. J. Bot. 83, 121–126. 10.1016/j.sajb.2012.08.007

[ref5] Chernyad’ev I. I. (2009). The protective action of cytokinins on the photosynthetic machinery and productivity of plants under stress (review). Appl. Biochem. Microbiol. 45, 351–362. 10.1134/S0003683809040012 19764606

[ref6] Connor E. F. Bartlett L. O’Toole S. Byrd S. Biskar K. Orozco J. (2012). The mechanism of gall induction makes galls red. Arthropod-Plant Inte. 6, 489–495. 10.1007/s11829-012-9210-7

[ref7] Dorchin N. Cramer M. D. Hoffmann J. H. (2006). Photosynthesis and sink activity of wasp-induced galls in *Acacia pycnantha*. Ecology 87, 1781–1791. 10.1890/0012-9658(2006)87[1781:PASAOW]2.0.CO;2, PMID: 16922327

[ref8] Eitle M. W. Griesser M. Vankova R. Dobrev P. Aberer S. Forneck A. (2019). Grape phylloxera (*D. vitifoliae*) manipulates SA/JA concentrations and signalling pathways in root galls of *Vitis* spp. Plant Physiol. Biochemist 144, 85–91. 10.1016/j.plaphy.2019.09.024 31561201

[ref9] Fay P. A. Hartnett D. C. Knapp A. K. (1996). Plant tolerance of gall-insect attack and gall-insect performance. Ecology 77, 521–534. 10.2307/2265627

[ref10] Firn R. D. (1989). Phenolic biosynthesis, leaf damage, and insect herbivory in birch (*Betula pendula*). J. Chem. Ecol. 15, 275–283. 10.1007/BF02027789, PMID: 24271442

[ref11] Gerchman Y. Lev-Yadun S. Inbar M. (2013). Red gall pigmentation: cytokinin stimulation is not everything. Arthropod-Plant Inte. 7, 335–337. 10.1007/s11829-013-9248-1

[ref12] Guimarães R. Barros L. Carvalho A. M. Sousa M. J. Morais J. S. Ferreira I. C. F. R. (2009). Aromatic plants as a source of important phytochemicals: vitamins, sugars and fatty acids in *Cistus ladanifer*, *Cupressus lusitanica* and *Eucalyptus gunnii* leaves. Ind. Crop. Prod. 30, 427–430. 10.1016/j.indcrop.2009.08.002

[ref13] Hilker M. Fatouros N. E. (2016). Resisting the onset of herbivore attack: plants perceive and respond to insect eggs. Curr. Opin. Plant Biol. 32, 9–16. 10.1016/j.pbi.2016.05.003, PMID: 27267276

[ref14] Hirano T. Kimura S. Sakamoto T. Okamoto A. Nakayama T. Matsuura T.. (2020). Reprogramming of the developmental program of *Rhus javanica* during initial stage of gall induction by *Schlechtendalia chinensis*. Front. Plant Sci. 11:471. 10.3389/fpls.2020.00471, PMID: 32499792 PMC7243852

[ref15] Howe G. A. Jander G. (2008). Plant immunity to insect herbivores. Annu. Rev. Plant Biol. 59, 41–66. 10.1146/annurev.arplant.59.032607.092825, PMID: 18031220

[ref16] Huang M. Y. Huang W. D. Chou H. M. Chen C. C. Chen P. J. Chang Y. T.. (2015). Structural, biochemical, and physiological characterization of photosynthesis in leaf-derived cup-shaped galls on *Litsea acuminata*. BMC Plant Biol. 15:61. 10.1186/s12870-015-0446-0, PMID: 25849781 PMC4351895

[ref17] Huang M. Y. Lin K. H. Yang M. M. Chou H. M. Yang C. M. Chang Y. T. (2011). Chlorophyll fluorescence, spectral properties, and pigment composition of galls on leaves of *Machilus thunbergii*. Int. J. Plant Sci. 172, 323–329. 10.1086/658157

[ref18] Iqbal N. Khan N. A. Ferrante A. Trivellini A. Francini A. Khan M. I. R. (2017). Ethylene role in plant growth, development and senescence: interaction with other phytohormones. Front. Plant Sci. 8:475. 10.3389/fpls.2017.00475 28421102 PMC5378820

[ref19] Kai S. Kumashiro S. Adachi S. Suzuki Y. Shiomi Y. Matsunaga K. (2017). Life history of *Stenopsylla nigricornis* (Hemiptera: Psylloidea: Triozidae) and phytohormones involved in its gall induction. Arthropod-Plant Inte. 11, 1–10. 10.1007/s11829-016-9470-8

[ref20] Kojima M. Kamada-Nobusada T. Komatsu H. Takei K. Kuroha Mizutani M.. (2009). Highly sensitive and high-throughput analysis of plant hormones using MS-probe modification and liquid chromatography–tandem mass spectrometry: an application for hormone profiling in *Oryza sativa*. Plant Cell Physiol. 50, 1201–1214. 10.1093/pcp/pcp057, PMID: 19369275 PMC2709547

[ref21] Kuang A. Peterson C. M. Dute R. R. (1992). Leaf abscission in soybean: cytochemical and ultrastructural changes following benzylaminopurine treatment. J. Exp. Bot. 43, 1611–1619. 10.1093/jxb/43.12.1611

[ref22] Kutsukake M. Uematsu K. Fukatsu T. (2019). Plant manipulation by gall-forming social aphids for waste management. Front. Plant Sci. 10:933. 10.3389/fpls.2019.00933, PMID: 31396247 PMC6664026

[ref23] Larson K. C. Whitham T. G. (1997). Competition between gall aphids and natural plant sinks: plant architecture affects resistance to galling. Oecologia 109, 575–582. 10.1007/s004420050119, PMID: 28307342

[ref24] Li X. Q. Liu Y. Z. Guo W. F. Solanki M. K. Yang Z. D. Xiang Y.. (2017). The gall wasp *Leptocybe invasa* (Hymenoptera: Eulophidae) stimulates different chemical and phytohormone responses in two *Eucalyptus varieties* that vary in susceptibility to galling. Tree Physiol. 37, 1208–1217. 10.1093/treephys/tpx098, PMID: 28938058

[ref25] Liu S. D. Ren S. Zheng J. Teng S. J. Fan H. J. Dai Y. Y. (2011). Study on distribution and rules of piercing-sucking insects in Jilin province (in Chinese). Forest. Sci. Tech. Info. 43, 9–11.

[ref26] Mapes C. C. Davies P. J. (2001). Cytokinins in the ball gall of *Solidago altissima* and in the gall forming larvae of *Eurosta solidaginis*. New Phytol. 151, 203–212. 10.1046/j.1469-8137.2001.00158.x 33873383

[ref27] McAtee P. Karim S. Schaffer R. David K. (2013). A dynamic interplay between phytohormones is required for fruit development, maturation, and ripening. Front. Plant Sci. 4:79. 10.3389/fpls.2013.00079, PMID: 23616786 PMC3628358

[ref28] Monica S. Rajat S. Anuradha S. Pankaj K. Ankita M. Sanjay J.. (2016). Comparative analysis of phenolic compound characterization and their biosynthesis genes between two diverse bread wheat (*Triticum aestivum*) varieties differing for chapatti (unleavened flat bread) quality. Front. Plant Sci. 7:1870. 10.3389/fpls.2016.01870, PMID: 28018403 PMC5156688

[ref29] Musser R. O. Hummusser S. M. Eichenseer H. Peiffer M. Ervin G. Murphy J. B.. (2002). Herbivory: caterpillar saliva beats plant defences. Nature 416, 599–600. 10.1038/416599a, PMID: 11948341

[ref30] Nandi S. K. Letham D. S. Palni L. M. S. Wong O. C. Summons R. E. (1989). 6-Benzylaminopurine and its glycoside as naturally occurring cytokinin. Plant Sci. 61, 189–196. 10.1016/0168-9452(89)90223-9

[ref31] Naseem M. Wolfling M. Dandekar T. (2014). Cytokinins for immunity beyond growth, galls and green islands. Trends Plant Sci. 19, 481–484. 10.1016/j.tplants.2014.04.001, PMID: 24794463

[ref32] Oliveira D. C. Isaias R. M. S. Fernandes G. W. Ferreira B. G. Carneiro R. G. S. Fuzaro L. (2016). Manipulation of host plant cells and tissues by gall-inducing insects and adaptive strategies used by different feeding guilds. J. Insect Physiol. 84, 103–113. 10.1016/j.jinsphys.2015.11.012, PMID: 26620152

[ref33] Oliveira D. C. Moreira A. Isaias R. M. S. Martini V. Rezende U. C. (2017). Sink status and photosynthetic rate of the leaflet galls induced by *Bystracoccus mataybae* (Eriococcidae) on *Matayba guianensis* (Sapindaceae). Front. Plant Sci. 8:1249. 10.3389/fpls.2017.01249, PMID: 28791033 PMC5522869

[ref34] Sáenz L. Jones L. H. Oropeza C. Vlácil D. Strnad M. (2003). Endogenous isoprenoid and aromatic cytokinins in different plant parts of *Cocos nucifera* (L.). Plant Growth Regul. 39, 205–215. 10.1023/A:1022851012878

[ref35] Samsone I. Andersone U. Ievinsh G. (2011). Gall midge *Rhabdophaga rosaria*-induced rosette galls on *Salix*: morphology, photochemistry of photosynthesis and defense enzyme activity. Env. Exp. Biol. 9, 29–36.

[ref36] Samsone I. Andersone U. Ievinsh G. (2012). Variable effect of arthropod-induced galls on photochemistry of photosynthesis, oxidative enzyme activity and ethylene production in tree leaf tissues. Env. Exp. Biol. 10, 15–26.

[ref37] Stahl E. Hilfiker O. Reymond P. (2018). Plant-arthropod interactions: who is the winner? Plant J. 93, 703–728. 10.1111/tpj.13773, PMID: 29160609

[ref38] Stone G. N. Schonrogge K. (2003). The adaptive significance of insect gall morphology. Trends Ecol. Evol. 18, 512–522. 10.1016/S0169-5347(03)00247-7

[ref39] Straka J. R. Hayward A. R. Emery R. J. N. (2010). Gall-inducing *Pachypsylla celtidis* (Psyllidae) infiltrate hackberry trees with high concentrations of phytohormones. J. Plant Interact. 5, 197–203. 10.1080/17429145.2010.484552

[ref40] Takei M. Yoshida S. Kawai T. Hasegawa M. Suzuki Y. (2015). Adaptive significance of gall formation for a gall-inducing aphids on Japanese elm trees. J. Insect Physiol. 72, 43–51. 10.1016/j.jinsphys.2014.11.006, PMID: 25437243

[ref41] Tooker J. F. Helms A. M. (2014). Phytohormone dynamics associated with gall insects, and their potential role in the evolution of the gall-inducing habit. J. Chem. Ecol. 40, 742–753. 10.1007/s10886-014-0457-6, PMID: 25027764

[ref42] Wang F. Sha J. Chen Q. Xu X. Zhu Z. Ge S.. (2020). Exogenous abscisic acid regulates distribution of ^13^C and ^15^N and anthocyanin synthesis in ‘Red Fuji’ apple fruit under high nitrogen supply. Front. Plant Sci. 10:1738. 10.3389/fpls.2019.01738, PMID: 32063908 PMC6997889

[ref43] Wellburn A. R. (1994). The spectral determination of chlorophylls *a* and *b*, as well as total carotenoids, using various solvents with spectrophotometers of different resolution. J. Plant Physiol. 144, 307–313. 10.1016/S0176-1617(11)81192-2

[ref44] Yamaguchi H. Tanaka H. Hasegawa M. Tokuda M. Asami T. Suzuki Y. (2012). Phytohormones and willow gall induction by a gall-inducing sawfly. New Phytol. 196, 586–595. 10.1111/j.1469-8137.2012.04264.x, PMID: 22913630

[ref45] Yang C. M. Yang M. M. Huang M. Y. Hsu J. M. Jane W. N. (2007). Life time deficiency of photosynthetic pigment-protein complexes CP1, A1, AB1, and AB2 in two cecidomyiid galls derived from *Machilus thunbergii* leaves. Photosynthetica 45, 589–593. 10.1007/s11099-007-0101-6

[ref46] Yu Z. J. Qi H. A. Jiang Z. l. Li G. W. (1998). Three-stage and sequential sampling techniques for the emigrant form of *Adelges laricis* Vall (in Chinese). *J*. Beijing Forest. Uni. 20, 47–51.

[ref47] Zhou W. Jia M. Y. Zhang G. C. Sun J. Li Q. L. Wang X. L.. (2020). Up-regulation of phenylpropanoid biosynthesis system in peach species by peach aphids produces anthocyanins that protect the aphids against UVB and UVC radiation. Tree Physiol. 10.1093/treephys/tpaa132, PMID: [Epub ahead of print] 33079182

[ref48] Zhu-Salzman K. Zeng R. S. (2015). Insect response to plant defensive protease inhibitors. Annu. Rev. Entomol. 60, 233–252. 10.1146/annurev-ento-010814-020816, PMID: 25341101

[ref49] Ziegler J. Qwegwer J. Schubert M. Erickson J. L. Schattat M. Burstenbinder K.. (2014). Simultaneous analysis of apolar phytohormones and 1-aminocyclopropan-1-carboxylic acid by high performance liquid chromatography/electrospray negative ion tandem mass spectrometry via 9-fluorenylmethoxycarbonyl chloride derivatization. J. Chromatogr. A 1362, 102–109. 10.1016/j.chroma.2014.08.029, PMID: 25160953

[ref50] Žurovcová M. Havelka J. Starý P. VěChtová P. Chundelová D. Jarošová A. (2010). "DNA barcoding" is of limited value for identifying adelgids (Hemiptera: Adelgidae) but supports traditional morphological taxonomy. Eur. J. Entomol. 107, 147–156. 10.14411/eje.2010.020

