# Supplementary material for: Phytohormones Regulate Both “Fish Scale” Galls and Cones on Picea koraiensis
Source: Front Plant Sci. 2020 Nov 27;11:580155. doi: 10.3389/fpls.2020.580155 (PMC7729011; doi:10.3389/fpls.2020.580155)
Supplement: Supplementary file 1 [file Table_1.doc]

**Phytohormones regulate both “fish scale” galls and cones on *Picea koraiensis***

**Supplmentary materials**

Mingyue Jia^1^, Qilong Li^1^, Juan Hua^1^, Jiayi Liu^1^, Wei Zhou^1^, Bo Qu^1,2^ and Shihong Luo^1,2*^

^1^College of Bioscience and Biotechnology, Shenyang Agricultural University, Shenyang, 110866, Liaoning Province, China

^2^Key Laboratory of Biological Invasions and Global Changes, Shenyang, 110161, Liaoning Province, China

*** Correspondence:**Corresponding Author: Shihong Luo
**luoshihong@syau.edu.cn**

**
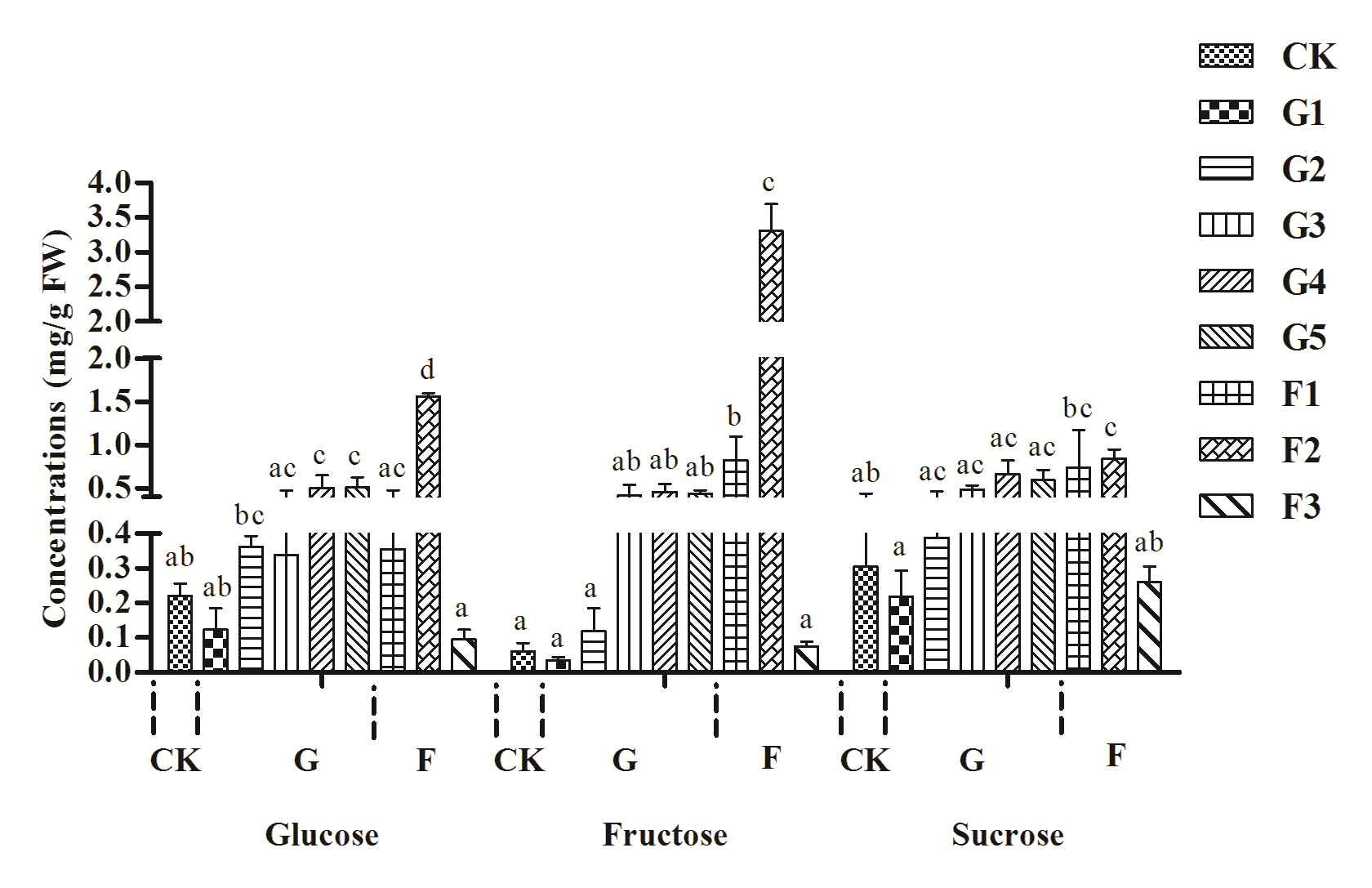
**

**Figure S1. Concentrations of glucose, fructose, and sucrose in gall tissues and cones.** CK= normal branches. G1−G5 represent the different developmental stages of gall tissues and F1−F3 represent different developmental stages of cones. Mean differences were compared using one-way ANOVA with Tukey test. The different small letters (a, b, c, d) represent significant difference at 0.05 level. These results shown represent the average ± SD.

**Table S1. Optimized MS conditions^a^**

| No. | Analyte | Mode | MRM transition | Q1 Pre  Bais (V) | CE (V) | Q1 Pre  Bais (V) |
| --- | --- | --- | --- | --- | --- | --- |
| 1 | GA_1_ | ESI- | 347.05>229.10 | 12 | 31 | 22 |
| 2 | GA_3_ | ESI- | 345.05>143.05 | 12 | 28 | 26 |
| 3 | GA_4_ | ESI- | 331.05>213.10 | 22 | 32 | 21 |
| 4 | GA_7_ | ESI- | 329.20>223.20 | 23 | 18 | 23 |
| 5 | GA_9_ | ESI- | 315.05>271.00 | 11 | 21 | 27 |
| 6 | GA_12_ | ESI- | 331.05>201.05 | 20 | 45 | 28 |
| 7 | GA_19_ | ESI- | 361.05>133.00 | 24 | 43 | 24 |
| 8 | GA_24_ | ESI- | 345.05>213.15 | 24 | 43 | 24 |
| 9 | GA_53_ | ESI- | 347.10>189.10 | 12 | 38 | 17 |
| 10 | *d_2_*-GA_1_ | ESI- | 349.20>275.20 | 22 | 25 | 28 |
| 11 | *d_2_*-GA_4_ | ESI- | 333.05>215.10 | 22 | 33 | 21 |
| 12 | IAA | ESI+ | 176.00>130.00 | -19 | -15 | -22 |
| 13 | IPA | ESI+ | 189.85>130.10 | -20 | -16 | -24 |
| 14 | *t*Z | ESI+ | 220.10>136.05 | -15 | -17 | -20 |
| 15 | BAP | ESI+ | 225.95>91.25 | -25 | -25 | -20 |
| 16 | iP | ESI+ | 203.95>136.20 | -14 | -18 | -27 |
| 17 | ABA | ESI- | 263.10>153.10 | 20 | 12 | 28 |
| 18 | *d_6_*-ABA | ESI- | 269.20>159.20 | 17 | 11 | 28 |
| 19 | JA | ESI- | 209.25>59.15 | 15 | 11 | 15 |
| 20 | PTH-ACC | ESI+ | 219.00>98.15 | -25 | -21 | -19 |
| 21 | SA | ESI- | 137.20>93.20 | 10 | 15 | 16 |
| 22 | MeSA | ESI- | 151.00>92.10 | 29 | 23 | 25 |
| 23 | *p*HBA | ESI- | 137.00>93.15 | 15 | 16 | 13 |
| 24 | *p*HCA | ESI- | 162.85>119.20 | 20 | 15 | 11 |
| 25 | *m*HBA | ESI- | 137.00>93.15 | 15 | 16 | 13 |
| 26 | *o*HCA | ESI- | 162.90>119.20 | 28 | 14 | 10 |
| 27 | *m*HCA | ESI- | 162.90>117.15 | 18 | 25 | 10 |
| 28 | BA | ESI- | 121.20>77.05 | 14 | 14 | 10 |
| 29 | *t*CA | ESI- | 147.05>103.20 | 17 | 15 | 15 |

^a^ GA, gibberellins; IAA, indole-3-acetic acid; IPA, 3-indolepropionic acid; IBA, 3-indolebutyric acid; *t*Z, *trans*-zeatin; BAP, 6-benzylaminopurine; iP, isopentenyladenine; ABA, abscisic acid; JA, jasmonic acid; PTH-ACC, phenylthiohydantoin-1-Aminocyclopropane-1-carboxylate; SA, salicylic acid; MeSA, methyl salicylate; *p*HBA, *p*-hydroxybenzoic acid; *p*HCA, *p*-hydroxycinnamic acid; *m*HBA, *m*-hydroxybenzoic acid; *o*HCA, *o*-hydroxycinnamic acid; *m*HCA, *m*-hydroxycinnamic acid; BA, benzoic acid; *t*CA, *trans*-cinnamic acid.

**Table S2.** Calibration equations for the analytes.

| No. | Analyte | Calibration equation | Linear range (μg/mL) | R^2^ value |
| --- | --- | --- | --- | --- |
| 1 | GA_1_ | Y=(1.99×10^-5)^X-0.0102 | 0.001−0.5 | 0.9935 |
| 2 | GA_3_ | Y = (3.25×10^-6^)X-0.0030 | 0.001−0.5 | 0.9994 |
| 3 | GA_4_ | Y = (4.99×10^-6^)X-0.0077 | 0.001−0.5 | 0.9986 |
| 4 | GA_7_ | Y=(2.35×10^-5^)X-0.0839 | 0.001−0.5 | 0.9933 |
| 5 | GA_9_ | Y=(9.54×10^-7^)X-8.897×10^-3^ | 0.001−0.5 | 0.9929 |
| 6 | GA_12_ | Y=(2.12×10^-5^)X-0.0023 | 0.001−0.5 | 0.9933 |
| 7 | GA_19_ | Y = (2.13×10^-5^)X+0.0034 | 0.001−0.5 | 0.9972 |
| 8 | GA_24_ | Y=(2.92×10^-5^)X-0.0011 | 0.005−0.5 | 0.9952 |
| 9 | GA_53_ | Y = (6.12×10^-6^)X+0.0002 | 0.001−0.5 | 0.9950 |
| 10 | *d*_2_-GA_4_ | Y=(6.45×10-^4^)X-0.2063 | 0.01−1 | 0.9930 |
| 11 | IAA | Y=(2.03×10^-7^)X-4.50×10^-2^ | 0.01−1 | 0.9993 |
| 12 | IPA | Y = (1.31×10^-7^)X+2.577×10^-2^ | 0.01−1 | 0.9984 |
| 13 | *t*Z | Y = (1.17×10^-7^)X-2.918×10^-2^ | 0.005−1 | 0.9984 |
| 14 | BAP | Y = (4.85×10^-6^)X-0.0172 | 0.005−1 | 0.9985 |
| 15 | iP | Y = (1.39×10^-6^)X+5.75×10^-3^ | 0.001−1 | 0.9990 |
| 16 | ABA | Y = (8.52×10^-6^)X+0.0083 | 0.005−1 | 0.9987 |
| 17 | JA | Y = (2.50×10^-7^)X-0.0247 | 0.001−1 | 0.9986 |
| 18 | PTH-ACC | Y = (2.54×10^-6^)X-1.7432 | 0.0005−0.5 | 0.9993 |
| 19 | SA | Y = (5.77×10^-8^)X-0.0038 | 0.001−1 | 0.9900 |
| 20 | MeSA | Y = (5.14×10^-7^)X-0.0077 | 0.005−1 | 0.9984 |
| 21 | *p*HBA | Y = (1.29×10^-7^)X-0.0190 | 0.001−0.5 | 0.9989 |
| 22 | *p*HCA | Y = (6.00×10^-8^)X-0.0103 | 0.001−0.5 | 0.9982 |
| 23 | *m*HBA | Y = (8.86×10^-7^)X+0.0824 | 0.001−0.5 | 0.9983 |
| 24 | *o*HCA | Y = (2.74×10^-7^)X+0.0045 | 0.001−0.5 | 0.9965 |
| 25 | *m*HCA | Y = (9.17×10^-8^)X+0.0029 | 0.001−0.5 | 0.9996 |
| 26 | BA | Y = (1.27×10^-5^)X-0.0262 | 0.001−0.5 | 0.9982 |
| 27 | *t*CA | Y = (7.36×10^-6^)X+0.1340 | 0.001−0.5 | 0.9989 |
